# Reliability of a New Digital Tool for Photographic Analysis in Quantifying Body Asymmetry in Scoliosis

**DOI:** 10.3390/jcm13072114

**Published:** 2024-04-05

**Authors:** Javier Pizones, Lucía Moreno-Manzanaro, Anika Pupak, Susana Núñez-Pereira, Daniel Larrieu, Louis Boissiere, Sarah Richner-Wunderlin, Markus Loibl, Tais Zulemyan, Altug Yücekul, Sara Zgheib, Yann Philippe Charles, Dong-Gune Chang, Frank Kleinstueck, Ibrahim Obeid, Ahmet Alanay, Francisco Javier Sánchez Pérez-Grueso, Ferran Pellisé, on behalf of the ESSG

**Affiliations:** 1 Spine Unit, Department of Orthopedic Surgery, Hospital Universitario La Paz, 28046 Madrid, Spain; luciam409@gmail.com (L.M.-M.); perezgrueso@gmail.com (F.J.S.P.-G.); 2 Spine Research Unit, Vall d’Hebron Institute of Research, 08035 Barcelona, Spain; anika.pupak@vhir.org; 3 Spine Surgery Unit, Hospital Universitario Vall d’Hebron, 08035 Barcelona, Spain24361fpu@gmail.com (F.P.); 4 Spine Surgery Unit, Pellegrin University Hospital, 33076 Bordeaux, France; larrieudaniel@gmail.com (D.L.); ibrahim.obeid@gmail.com (I.O.); 5 Department of Orthopedics, Schulthess Klinik, 8008 Zurich, Switzerlandmarkus.loibl@gmail.com (M.L.); frank.kleinstueck@kws.ch (F.K.); 6 Department of Orthopedics and Traumatology, Acibadem Mehmet Ali Aydinlar University, 34752 Istanbul, Turkey; tais.zulemyan@gmail.com (T.Z.); ayucekul@gmail.com (A.Y.); aalanay@gmail.com (A.A.); 7 Spine Surgery Unit, University Hospital Strasbourg, 67000 Strasbourg, France; sara.zgheib@chru-strasbourg.fr (S.Z.); yannphilippe.charles@chru-strasbourg.fr (Y.P.C.); 8 Department of Orthopedic Surgery, Inje University Sanggye Paik Hospital, College of Medicine, Inje University, 01757 Seoul, Republic of Korea; dgchangmd@gmail.com

**Keywords:** clinical photography, scoliosis assessment, body asymmetry, photogrammetry, reliability study, intraclass correlation coefficients

## Abstract

**Background:** Advancements in non-ionizing methods for quantifying spinal deformities are crucial for assessing and monitoring scoliosis. In this study, we analyzed the observer variability of a newly developed digital tool for quantifying body asymmetry from clinical photographs. **Methods:** Prospective observational multicenter study. Initially, a digital tool was developed using image analysis software, calculating quantitative measures of body asymmetry. This tool was integrated into an online platform that exports data to a database. The tool calculated 10 parameters, including angles (shoulder height, axilla height, waist height, right and left waistline angles, and their difference) and surfaces of the left and right hemitrunks (shoulders, waists, pelvises, and total). Subsequently, an online training course on the tool was conducted for twelve observers not involved in its development (six research coordinators and six spine surgeons). Finally, 15 standardized back photographs of adolescent idiopathic scoliosis patients were selected from a multicenter image bank, representing various clinical scenarios (different age, gender, curve type, BMI, and pre- and postoperative images). The 12 observers measured the photographs at two different times with a three-week interval. For the second round, the images were randomly mixed. Inter- and intra-observer variabilities of the measurements were analyzed using intraclass correlation coefficients (ICCs), and reliability was measured by the standard error of measurement (SEM). Group comparisons were made using Student’s *t*-test. **Results:** The mean inter-observer ICC for the ten measurements was 0.981, the mean intra-observer ICC was 0.937, and SEM was 0.3–1.3°. The parameter with the strongest inter- and intra-observer validity was the difference in waistline angles 0.994 and 0.974, respectively, while the highest variability was found with the waist height angle 0.963 and 0.845, respectively. No test–retest differences (*p* > 0.05) were observed between researchers (0.948 ± 0.04) and surgeons (0.925 ± 0.05). **Conclusion:** We developed a new digital tool integrated into an online platform demonstrating excellent reliability and inter- and intra-observer variabilities for quantifying body asymmetry in scoliosis patients from a simple clinical photograph. The method could be used for assessing and monitoring scoliosis and body asymmetry without radiation.

## 1. Introduction

Adolescent idiopathic scoliosis (AIS), even if properly diagnosed and treated, can progress during growth, impacting patients’ qualities of life, regarding pain, disability, mental health, and self-image perception. Physical appearance in these patients is associated with psychological distress [1], and cosmetic concerns, especially waistline asymmetry [2,3], becomes one of the reasons for electing surgery [4]. It is, therefore, important to find a tool apart from clinical inspection that can objectively assess body appearance.

On the other hand, the diagnosis of any deviation of the spine, the assessment of the curve magnitude, and the detection of progression are mainly based on radiographic assessment. Even though attempts to decrease radiation have been made with the development of the EOS (low-dose bi-planar radiography) system [5], it is still poorly extended in spine referral centers, as installation and operation costs are still high. Radiation is cumulative as longitudinal follow-ups are generally required in this condition [6], and some reports are showing a greater incidence of tumors in this population when reaching adulthood [7]. Thus, advancements in non-ionizing methods for quantifying spinal deformities are crucial for assessing and monitoring scoliosis patients.

Several methods can measure body appearance and assess scoliosis in a non-radiation approach such as rasterstereography [8,9], ultrasonography [10,11], and surface topography (ST) systems (using photogrammetry [12,13], structured light [14], or laser scanning [15]). 

ST captures 3D images of the surface of patients’ torsos that are later analyzed with computer programs offering angles, volumes, areas, or distances that measure body asymmetry. Most ST measurements have shown satisfactory (good to excellent) reliability and validity [16], although some systems require the operator to manually place the marker points at the specified anatomical locations to increase the accuracy of the measurement. Still, the biggest drawback is that raw images need to be matched, processed, calibrated, landmarked, analyzed, and interpreted, which is usually a complex time-consuming process. Methods usually require expensive equipment and a trained operator. Thus, currently, ST is almost reserved for research projects and has limited clinical applications.

Another alternative is the use of clinical photographs. Measurement of body angles from photographs is considered the most accurate and rapid way to assess global posture quantitatively in a clinical setting [17]. It is a valid method to assess trunk asymmetry, it can discriminate among different curve patterns according to the Lenke classification [18], and it has the great advantage of being easy to obtain in daily practice [19]. Clinical photographs have shown consistency in image analysis and correlations with body self-perception, radiographic angular measurements, and quantifiable image scales (such as TAPS) [20,21]. The current limitation for its universal use is the lack of automatic software that can reliably measure these photographs [18,22]. 

In the present study, we aimed to analyze the reliability, reproducibility, and repeatability of a newly developed digital tool for quantifying body asymmetry from clinical photographs taken of adolescents with idiopathic scoliosis.

## 2. Materials and Methods

We conducted a prospective observational multicenter study using the bank of clinical photographs from AIS patients collected in the (European Spine Study Group (ESSG)) database. The study protocol was approved by the ethics committees of each site and followed the protocols described in the Helsinki Declaration for human-based research. All the participating patients and their parents gave prior consent to obtaining the clinical photographs and to their inclusion in the study. 

### 2.1. Inclusion Criteria 

Inclusion criteria were patients with AIS (10–17 years) with available clinical photographs taken from the back as a standard examination before elective surgery (main Cobb angle > 45°) and/or taken during the postoperative follow-up. Exclusion criteria were younger or older patients, non-idiopathic deformities, or those previously operated on.

### 2.2. Photograph Acquisition

Photographs were taken with the digital camera integrated into a common smartphone, and no specific direct lightning was used. Patients were told to adopt a relaxed standing position with a naked torso (boys), and girls were not asked to take off their bras. Pictures were taken 1 m away with the focus perpendicular to the mid part of the spine, no calibration mark was needed, and no anatomical landmark was externally added. Patients with long hair were asked to attain a ponytail to leave the neck free from hair. All patients were photographed from the rear (posterior or back view).

### 2.3. Designing the Digital Measuring Tool

In the first part of the project, a new digital tool was developed together with a computer engineer creating image analysis software able to calculate quantitative measures of body asymmetry from these clinical photographs of the back. This image analyzer was subsequently integrated into an online platform that automatically exported the calculated data into a database. The tool worked as follows.

**Figure 1:**
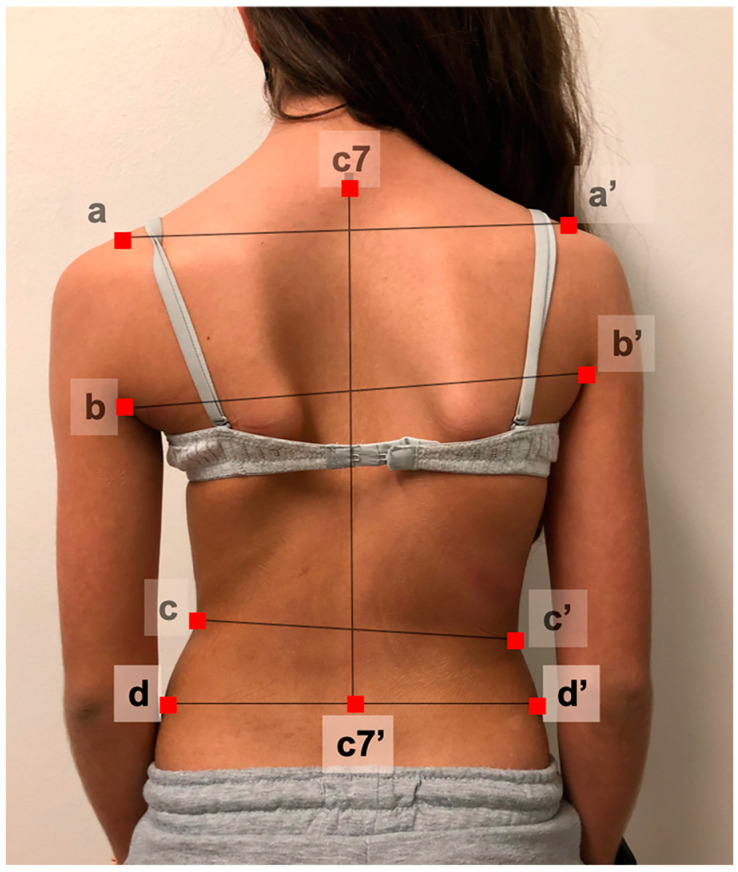
Anatomical landmarks: a and a’ shoulder points; b and b’ axilla points; c and c’ waist points; the C7 spinous process and C7′ (midpoint of a vertical line thrown from C7); and d, d’ iliac crest points.

Once the photograph was uploaded into the system, the software automatically checked image quality. Subsequently, 9 anatomical landmarks were selected by the evaluator (manually tagged with the computer mouse). In Figure 1, 2 shoulder points located at the endpoint of both acromions; 2 axilla points located at the most superior points of both posterior axillary folds; 2 waist points located at the ‘minimal waist’, which correspond to the narrowest portion of both waists; the C7 spinous process; and 2 iliac crests points located on the most external points of both iliac crests. The tool automatically created a series of lines intersecting these pairs of points and calculated 10 different parameters based on Matamalas et al. descriptions [23,24]. These parameters were defined among others by these cited authors as the most reliable measurements in digital photography to assess shoulder, trunk, and waist asymmetry in idiopathic scoliosis patients displaying angles and areas.

Regarding angles, we measured shoulder height angle (SHA), axilla height angle (AHA), waist height angle (WHA), left waistline angle (LWA), right waistline angle (RWA), and waistline angle difference (WAD). Values were assigned positive or negative according to the tilt direction. The right-hand thumb rule was used for this: looking at the individual from the back, a clockwise tilt was considered positive and an anti-clockwise tilt negative.

**Figure 2:**
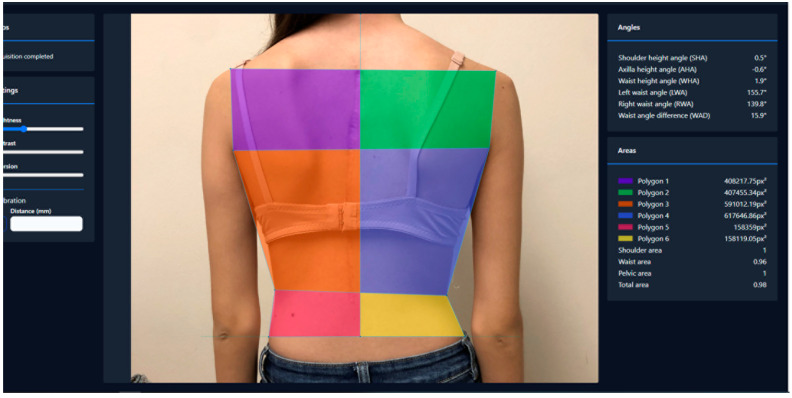
Example of the parameters obtained (angles and areas) with the digital tool assessing the clinical photograph of an AIS patient.

Following Bago et al. [18]’s research, several polygons were drawn, dividing the left and right back parts through the C7-plumbline and cranial to caudal areas by the horizontal lines drawn with the previous anatomical landmarks. Surfaces/areas were then measured by the system using square pixels, of the left and right hemitrunks. The shoulder area (SA), waist area (WA), pelvic area (PA), and a total area (TA) (total left vs. right differences) were finally calculated (Figure 2).

### 2.4. Observer Training

Subsequently, an online training course on the tool was conducted for twelve observers not involved in the image analyzer tool development. Observers were chosen from our multicenter group, so they were multinational with different ages and genders. Observers had different measurement skills, as six of them were research coordinators experienced in radiographic image software analyzers, and the other six were spine surgeons from our multicenter consortium.

### 2.5. Reliability, Reproducibility, and Repeatability Tests

Finally, from the 250 available standardized back photographs of adolescent idiopathic scoliosis patients in our multicenter image bank, 15 were selected, representing various clinical scenarios that included different ages, genders, curve types, BMIs, races, and presurgical and postoperative images. The proposed 12 observers (blinded to imaging selection) measured each photograph twice (two rounds). The first round took place two weeks after the training process, and the second round was after a three-week interval. For the second round, images were randomly mixed.

### 2.6. Statistical Analysis

Statistical analysis was carried out using SPSS software (version 20, SAS Institute Inc., Cary, NC, USA). Normality of the variables was tested using Kolmogorov–Smirnov test. The distribution of quantitative variables was given as mean and standard deviation. To determine observer variability of the photographic measures, inter-observer (measuring reproducibility) and intra-observer (measuring repeatability) variability of the measurements were analyzed using intraclass correlation coefficients (ICCs) with a 2-way mixed random model and a 95% confidence interval. To calculate reproducibility, the first round of measurements was used. ICC results were interpreted applying published criteria (0.5–0.75 moderate variability, 0.75–0.9 good, and >0.9 excellent) [25]. Reliability was not a measure of precision; it was the error of measurement not intrinsic to the method but the population. Thus, to assess the reliability of the tool, the standard error of measurement was calculated with the first round of values using the formula SEM = $SD\times \sqrt{1-ICC}$ [26]. Univariate analysis comparing researchers’ vs. surgeons’ performances was performed using the Student’s *t*-test. The significance threshold was set at 5% (*p* < 0.05).

## 3. Results

All 12 observers attended the online course where they were introduced to the digital tool and were taught how to identify the anatomical reference landmarks and the meaning of all 10 measurements. Two weeks after the training call, the first round of 15 photos was run by the 12 observers. Table 1 shows the mean values for each of the 10 parameters (all had a normal distribution) from the first round. All values are displayed with a dispersion estimate, and angular values show also the standard error of measurement (reliability), which in general was found to be between 0.3° and 1.3° (Table 1).

The mean inter-observer ICC for the ten measurements (including all angles and areas) calculated from the first round of observations was 0.981 (95%CI: 0.963–0.992) (Table 2), which means excellent agreement.

Table 3 shows the intra-rater results of each measure for every observer. The mean intra-observer ICC (comparing each observer with himself first vs. second rounds) was 0.937 (range-R = 0.779–0.988) (Table 4), meaning excellent agreement.

The parameter with the strongest inter- and intra-observer variabilities was the difference in waistline angles 0.994 (95%CI: 0.988–0.998) and 0.974 (R = 0.916–0.997), respectively. The highest variabilities for both inter- and intra-observer were found with the waist height angles 0.963 (95%CI: 0.929–0.986) and 0.845 (R = 0.535–0.987), respectively.

No test–retest differences (*p* > 0.05) were observed between researchers (0.948 ± 0.04) and surgeons (0.925 ± 0.05) (Table 5).

## 4. Discussion

Idiopathic scoliosis patients have esthetic concerns independently of the magnitude of the curve [27]. Some authors emphasize that shoulder balance and scapular and waistline asymmetries are the most important features to consider [28]. However, it is waistline asymmetry that mainly bothers patients [2,3]. Physical appearance seems to be associated with psychological distress [1], and it is the second most important reason for surgery both for patients and parents [4]. Therefore, one paramount step in scoliosis assessment is to have a good tool to analyze body asymmetry for the detection and early diagnosis of scoliosis, as well as monitoring its progression during follow-up visits. Visual examination can detect these asymmetries, but it does not offer an objective and reliable method of evaluation and comparison.

Radiographs are still the gold standard to diagnose and monitor scoliosis progression. However, they can only partially reflect the objective cosmetic appearance, underlying the importance of the clinical evaluation in AIS assessment [21,29]. Other non-ionizing alternatives exist, but are not widely spread yet and are mostly utilized for research purposes. Rasterstereography, is a non-invasive stereophotogrammetric surface measurement, whereby a slide projector projects horizontal lines onto the patient’s back, which are then photographed and analyzed [30]. Ultrasonography measures the angle formed between the lines drawn on the most tilted part of the bony prominence’s shadow. Most descriptions scan the spinous processes as an anatomical reference (laminae and transverse processes can also be assessed). It has the advantage of being performed upright. It has shown high reliability and validity, mainly the center of lamina (COL) method [31]. However, it is very operator-dependent, and it can lead to underestimation of the Cobb angle or overestimation of mild curves [32]. Additionally, poor image quality can be experienced when there is a thick muscle/fat layer or inappropriate contact of the transducer [31]. Some curves can be missed while some identified curves can be false due to standing posture variation [33]. And of course, it does not assess body appearance.

Several technologies based on surface topography (ST) are available to objectively measure body asymmetry, such as structured light, infrared, or laser scanning [34]. The information obtained is captured by cameras and then processed to create a 3D model of the torso [13]. More than angles, ST provides objective measurements of areas and volumes, able to describe a torso’s reliefs. Photogrammetry uses multiple cameras to capture images of the body from different angles [12]. Structured light projects a pattern of parallel fringes onto the body surface (Moiré technique) [35] and collects the distortion of those fringes that can give information about the distance (height) of the surface of the object away from a flat reference plane [8]. Laser scanning uses laser beams to scan the body’s surface, which can be performed by external devices (scanner) or integrated into a smartphone or tablet [13,15]. Surface analysis can help to document the external asymmetry associated with scoliosis and the cosmetic improvement obtained after surgery [36].

All these ST systems detect volumes, areas, deepnesses, and distances to measure trunk asymmetry. However, they usually need a complicated process of calibration, image matching, triangulation, surface generation, operability, and connection to a computer system where the matching of a lot of surface points needs to be computed. Thus, they are very complex to analyze and interpret and are expensive [13]. Although some methods have correlated surface topographic measurements with patient-reported outcome measures (TAPS, SRS-22), correlations are low [37] to moderate, 0.49–0.65 [13].

Fortin et al. analyzed all the methods available to assess posture in the clinical setting (radiographs, goniometer, inclinometer, clinical assessment, surface topography systems, computerized motion analysis systems, and video methods), concluding that the most promising technique to assess posture globally is the calculation of body angles on photographs [17]. Measurement of body angles and distances from photographs may be the most accurate and rapid way to assess global posture quantitatively [17]. It avoids the complexity of the existing methods in terms of needed material, image matching and calibration, operability, and practicality. However, photographic assessment lacks a reliable semiautomatic measurement system to perform the measurements [18,22], and this is what has been addressed in the present study.

We developed a digital tool to objectively measure clinical photographs. We used the back view to avoid intimacy concerns in female patients and used anatomical points and selected angular measurements to avoid the need for calibration when using linear measurements. The anatomical landmarks we chose were based on previous investigations. Matamalas et al. analyzed which photographic measures described in the literature were more adequate for assessment regarding reliability and validity compared to radiographic and self-reported scores [23], and we adapted Bago et al.’s principles [18] to divide the back in sectors.

The shoulder (SHA) and axilla (AHA) height angles are not collinear to assess the clinical balance of the shoulders, so we needed to measure both separately. SHA can be considered the standard parameter to evaluate shoulder balance in clinical photos, as it correlates with the Clavicle–Rib Intersection Angle (CRIA) and T1 tilt [23]. Although it does not correlate with the proximal thoracic curve Cobb, it is able to discriminate Lenke 2 curve types [18]. AHA is more related to deformity of the trunk, demonstrating inverse correlation with the main thoracic curve Cobb [20,23]. Waistline asymmetry, which seems to be a key factor in the patient’s perception of trunk deformity, is better depicted with the waist height angle (WHA). This angle describes the slope between waist creases. It correlates with the main thoracic curve Cobb and with patients’ perceptions measured with TAPS and SAQ [24]. The WHA can discriminate Lenke types 1 and 2 from the rest of types [18], and although we found it to be the least reliable from all the parameters in our series, the intraclass coefficient was still excellent at 0.96. Finally, the difference between right and left waistline angles (RLWADs) correlates with thoracolumbar/lumbar Cobb and the lowest end vertebra inclination [24]. In our study, it was the parameter that showed the strongest reproducibility and repeatability at 0.99.

Some studies analyzed photographic reliability measurements, obtaining between good and excellent ICC inter- and intra-observer variabilities: 0.79–0.96 (with SEM between 0.83 and 2.56°) [23,24], 0.80–0.93 [38], and 0.91–0.99 [21]. However, all these studies used manual methods to evaluate photographs, but none of them described a standardized process yet; nor have they succeeded in developing a versatile, reliable tool that achieves standardization of body asymmetry measurements.

The digital tool we developed sets the basis for the most adequate parameters to analyze in the photographic assessment of the back asymmetry of scoliotic patients. Photographs are taken with a simple digital camera without the need for complex devices, specific direct lightning, calibration, or landmarking. Patients can adopt a relaxed standing position. Once the photograph is uploaded to the system, the evaluator digitally marks the nine anatomical points (Figure 1), following an animated guideline available with the tool. The system will automatically provide 10 different parameters that measure trunk asymmetry (Figure 2): shoulder, axilla, and waist heights; waistline angles; and shoulder, waist, and pelvic hemitrunk surfaces.

The tool proved to be reliable (which relates measurement error to the true variability within the measurement sample [26]) with an SEM between 0.3° and 1.3°, depending on the measured angle. It was reproducible (which is the ability of different observers to come up with the same measurement [26]), with a mean inter-observer ICC of 0.981, and it was repeatable (the ability of the same observer to come up with the same result on a second measurement performed on the same sample [26]), showing a mean intra-observer ICC of 0.93.

If we compare our results in clinical photography with other existing methods of deformity assessment, we find that our method stands out above average. Reports on ST reliability vary depending on the method evaluated, ranging from coefficients of 0.85 using ST maps [39], 0.90 [40] with automated topographic calculation and with the DIERS ST system [41], to ICC values of 0.97 ± 0.2 and 0.90 ± 0.02 (intra- and inter-observer reliabilities, respectively) using the Scolioscan system [34]. If we analyze the traditional Adams forward bending test, we find similar results to ours, with SEMs of 1.9° (intra-observer) and 0.8° (inter-observer) [42] and reliability coefficients of 0.86–0.97 for intra- and inter-observer assessments [43]. To give a notion of the figures found in Cobb radiological measurements, the mean manual measurement error is 3° [44]. There is a study comparing the surgeon’s manual method with a semi-automated machine learning semantic segmentation network that shows ICCs for both above 0.96, with SEMs around 3° [45].

The study, however, has some limitations. We only assessed one plane (back view), although our database also contains images from the lateral and Adams’ views, and we know that scoliosis is a 3D deformity, all planes are affected by the condition. However, there are still no standardized photographic measures to tackle these other two views. The identification of anatomical landmarks was performed digitally by the observer with no external previous back marks. This might be seen as imprecise, however, it seemed to be accurate due to the very good validity we found in the analysis. Due to the multicentric design of the study, the risk for systematic observer effects due to variations in skills and adherence to the study protocol between observers could increase. During the training program, major emphasis was laid on the standardization of anthropometric measurements to decrease measurement error, and the digital tool came with a 10-step user’s guide to conduct new observers through the landmark process. It worked fine, as our final results showed similar reliability when comparing experienced-measuring researchers to novel-measuring surgeons. On the other hand, we were very careful in the study design to overcome the most common flaws of reliability studies such as [16] qualifications of the testers (prior training), blinded testers, or order variation between rounds. The tested photographs have a high variety of patient characteristics: ages, genders, races, BMIs, and nationalities. We did not evaluate the sensitivity and responsiveness of photography measurements in identifying scoliosis progression nor the impact of the patient’s sway or postural changes that might affect observer variability (both can be future projects to conduct). The developed tool assesses a static image in a cross-sectional approach, so it can only be used as a reference to assess body asymmetry but does not directly indicate the cause of this asymmetry. Similar to what happens in ST studies, correlations between clinical and radiological parameters are just moderate (<0.6) [24].

The next steps for further research should include the analysis of the tool variability regarding postural changes and to study of its sensitivity to change with curve progression or surgical intervention. We still lack objective tools to measure body appearance even though we know that this item is a concern for our patients. We only measure this impact with self-perceived numerical scales (such as SAQ, TAPS, and SRS-22) or clinically using the scoliometer in the forward bending test. Other methods of assessing body asymmetry exist but they are complex to manage and are not yet incorporated in our clinical arsenal (such as surface topography). Clinical photographs can help in this regard as an easy and accessible way of measuring trunk asymmetry to aid in curve assessment and perhaps in the future monitor deformity progression if proven to be sensitive to change and aid in surgical decision making.

## 5. Conclusions

We developed a new digital tool integrated into an online platform demonstrating excellent reliability and inter- and intra-observer variabilities for quantifying body asymmetry in scoliosis patients from a simple clinical photograph. The method is a radiation-free, quick, easy, inexpensive, and accessible approach that can be used for assessing and monitoring scoliosis without radiation.

## Figures and Tables

**Table 1:** Descriptive presentation of the measured angles.

	N	Minimum	Maximum	Mean ± SD	Median (IR)	SEM
SHA (°)	15	−7.5	2.43	−2.5 ± 2.6	−2.8 (3.83)	0.30
AHA (°)	15	−7.8	−0.85	−4.4 ± 2.2	−3.9 (3)	1.17
WHA (°)	15	−3.1	6.58	2.0 ± 3.0	1.7 (3.7)	0.57
LWA (°)	15	128.4	157.18	144.8 ± 9.5	145.9 (17.4)	0.95
RWA (°)	15	132.3	163.58	147.9 ± 8.1	146.7 (12.1)	1.3
WAD (°)	15	−35.2	24.88	−3.1 ± 14.9	−6.4 (21.8)	1.15
SA px^2^	15	0.80	1.14	0.94 ± 0.09	0.93 (0.1)	0.02
WA px^2^	15	0.52	0.93	0.71 ± 0.12	0.69 (0.2)	0.06
PA px^2^	15	0.45	1.69	0.83 ± 0.3	0.78 (0.3)	0.12
TA px^2^	15	0.64	1.06	0.81 ± 0.12	0.81 (0.2)	0.05

SD: standard deviation; IR: interquartile range; SEM: standard error of measurement; SHA: shoulder height angle; AHA: axilla height angle; WHA: waist height angle; LWA: left waistline angle; RWA: right waistline angle; WAD: waistline angle difference; SA: shoulder area; WA: waist area; PA: pelvic area; TA: total area; °: degrees; and px^2^: square pixels.

**Table 2:** Intraclass correlation coefficient for inter-observer differences.

Inter-Observer	Intraclass Correlation	95% Confidence Interval		F Test	
		Lower Bound	Higher Bound	Value	Sig
SHA	0.986	0.972	0.994	81.5	0.000
AHA	0.973	0.942	0.990	62.9	0.000
WHA	0.963	0.929	0.986	28.0	0.000
LWA	0.990	0.979	0.996	124.7	0.000
RWA	0.972	0.946	0.989	37.0	0.000
WAD	0.994	0.988	0.998	161.2	0.000
SA	0.976	0.951	0.991	56.8	0.000
WA	0.987	0.975	0.995	89.7	0.000
PA	0.986	0.973	0.995	77.3	0.000
TA	0.988	0.977	0.995	100	0.000
Total Mean	0.981	0.963	0.992		

SHA: shoulder height angle; AHA: axilla height angle; WHA: waist height angle; LWA: left waistline angle; RWA: right waistline angle; WAD: waistline angle difference; SA: shoulder area; WA: waist area; PA: pelvic area; and TA: total area.

**Table 3:** Specific intra-rater correlation coefficients for each measurement of each observer.

Observer	SHA	AHA	WHA	LWA	RWA	WAD	SA	WA	PA	TA
Obsv1	0.946	0.985	0.955	0.977	0.99	0.988	0.927	0.977	0.98	0.969
Obsv2	0.953	0.978	0.926	0.918	0.934	0.997	0.962	0.972	0.994	0.988
Obsv3	0.752	0.791	0.734	0.946	0.887	0.968	0.785	0.899	0.931	0.86
Obsv4	0.992	0.99	0.637	0.99	0.988	0.997	0.966	0.979	0.989	0.98
Obsv5	0.908	0.954	0.943	0.98	0.815	0.947	0.966	0.957	0.961	0.959
Obsv6	0.96	0.975	0.926	0.963	0.979	0.98	0.934	0.98	0.865	0.986
Obsv7	0.931	0.973	0.881	0.973	0.974	0.994	0.901	0.957	0.984	0.971
Obsv8	0.962	0.974	0.985	0.983	0.98	0.99	0.879	0.945	0.989	0.952
Obsv9	0.962	0.985	0.987	0.986	0.941	0.994	0.937	0.978	0.991	0.976
Obsv10	0.993	0.989	0.982	0.993	0.997	0.996	0.941	0.975	0.994	0.975
Obsv11	0.921	0.818	0.655	0.923	0.609	0.931	0.894	0.92	0.946	0.951
Obsv12	0.913	0.966	0.535	0.778	0.796	0.916	0.919	0.974	0.969	0.973

SHA: shoulder height angle; AHA: axilla height angle; WHA: waist height angle; LWA: left waistline angle; RWA: right waistline angle; WAD: waistline angle difference; SA: shoulder area; WA: waist area; PA: pelvic area; and TA: total area.

**Table 4:** Intraclass correlation coefficient for intra-observer differences.

Variable	Mean	Standard Deviation	Range	
			Minimum	Maximum
SHA	0.93	0.06	0.752	0.993
AHA	0.95	0.06	0.791	0.990
WHA	0.84	0.16	0.535	0.987
LWA	0.95	0.06	0.778	0.993
RWA	0.91	0.12	0.609	0.997
WAD	0.97	0.03	0.916	0.997
SA	0.92	0.05	0.785	0.966
WA	0.96	0.03	0.899	0.980
PA	0.97	0.04	0.865	0.994
TA	0.96	0.03	0.860	0.988
Total Mean	0.937	0.06	0.779	0.988

SHA: shoulder height angle; AHA: axilla height angle; WHA: waist height angle; LWA: left waistline angle; RWA: right waistline angle; WAD: waistline angle difference; SA: shoulder area; WA: waist area; PA: pelvic area; and TA: total area.

**Table 5:** Comparison between researchers and surgeons regarding intra-observer intraclass correlation.

Variable	Researchers	Surgeons	Sig
SHA	0.96 ± 0.03	0.90 ± 0.08	0.126
AHA	0.98 ± 0.01	0.92 ± 0.09	0.154
WHA	0.83 ± 0.19	0.86 ± 0.13	0.784
LWA	0.94 ± 0.08	0.96 ± 0.02	0.479
RWA	0.94 ± 0.08	0.87 ± 0.14	0.283
WAD	0.98 ± 0.03	0.97 ± 0.03	0.609
SA	0.93 ± 0.03	0.90 ± 0.63	0.303
WA	0.97 ± 0.01	0.95 ± 0.03	0.151
PA	0.97 ± 0.05	0.96 ± 0.02	0.961
TA	0.97 ± 0.01	0.95 ± 0.44	0.186
Total Mean	0.948 ± 0.04	0.925 ± 0.05	0.410

SHA: shoulder height angle; AHA: axilla height angle; WHA: waist height angle; LWA: left waistline angle; RWA: right waistline angle; WAD: waistline angle difference; SA: shoulder area; WA: waist area; PA: pelvic area; and TA: total area.

## Data Availability

The data that support the findings of this study are available on request from the corresponding author [JP].
